# Deep learning for cardiac MRI: performance evidence and barriers to clinical integration. A Systematic Review and Meta-Analysis

**DOI:** 10.1093/ehjimp/qyag045

**Published:** 2026-03-16

**Authors:** Fatemah Aladwani, Alessandro Perelli, Ify Mordi, Faisel Khan

**Affiliations:** Department of Cardiovascular Research, School of Medicine, University of Dundee, Dundee DD1 9SY, UK; Jaber Alahmad Hospital, Ministry of Health, South Surra 46300, Kuwait; Division of Biomedical Engineering, School of Science and Engineering, University of Dundee, Dundee DD1 4HN, UK; School of Cardiovascular and Metabolic Health, College of Medicine, Veterinary and Life Sciences, University of Glasgow, Glasgow G12 8TA, UK; Department of Cardiovascular Research, School of Medicine, University of Dundee, Dundee DD1 9SY, UK; Department of Cardiovascular Research, School of Medicine, University of Dundee, Dundee DD1 9SY, UK

**Keywords:** deep learning (DL), CMR imaging, image segmentation, diagnosis, prediction, cardiovascular disease (CVD)

## Abstract

**Aims:**

This systematic review and meta-analysis aimed to evaluate the current evidence on the use of deep learning in cardiac magnetic resonance imaging, focusing on image segmentation, prediction, and diagnosis.

**Methods and results:**

A systematic search of Medline, Web of Science, Embase, and Scopus identified studies published between 2020 and 2025. Eligible studies comprised deep learning-based segmentation, prediction, or diagnosis of cardiac magnetic resonance images. MetaDisc version 1.4 was used for statistical analysis, with a *P* < 0.05 and an I^2^ ≥ 75% used as the thresholds for statistical significance and high heterogeneity, respectively. From 1510 retrieved articles, 62 studies met the inclusion criteria, and 12 studies were included in the meta-analysis. Most studies targeted segmentation (*n* & 45), with fewer addressing diagnosis (*n* & 9), and prediction (*n* & 28). Supervised learning predominated (91.94%), and U-Net was the most common architecture (70.97%). Mean Dice score (15 studies) was 0.91 ± 0.03, whereas mean Hausdorff distance (six studies) was 8.99 ± 6.45 mm. Diagnosis and prediction achieved pooled sensitivity of 0.94 (95% CI: 0.92–0.96), specificity of 0.91 (95% CI: 0.89–0.93), and AUC of 0.9831, indicating excellent discriminative ability. Segmentation models reached pooled sensitivity of 1.00 (95% CI: 0.99–1.00) and specificity of 0.98 (95% CI: 0.98–0.99). The AUC from the SROC analysis was 0.9940, confirming exceptional segmentation accuracy.

**Conclusion:**

Deep learning models show excellent performance in cardiac magnetic resonance segmentation and diagnosis, often matching or exceeding manual analysis, indicating strong potential for clinical adoption.

This systematic review was registered in the International Prospective Register of Systematic Reviews (PROSPERO) with the registration number CRD42023439659.

## Introduction

Cardiovascular disease (CVD) remains the leading global cause of mortality, with over 500 million prevalent cases and 18.6 million deaths in 2019.^1^ Early diagnosis is crucial for reducing CVD-related mortality and providing timely intervention. Cardiac function is primarily affected in all types of CVD. Several imaging modalities are used for cardiac function analysis, including computed tomography (CT), ultrasound, and magnetic resonance imaging (MRI).^2^ However, cardiac MRI (CMR) has emerged as the primary modality for diagnosing various types of cardiomyopathies, including ischaemic cardiomyopathy, dilated cardiomyopathy, hypertrophic cardiomyopathy, and tachycardia-induced cardiomyopathy.^3,4^

CMR can be used for the precise quantification of heart chamber dimensions, function, and volume, which are important prognostic indicators.^5^ CMR helps diagnose CVD by deriving cardiac indices, such as end-diastolic volume (EDV) and end-systolic volume (ESV).^6^ However, the widespread clinical implications of CMR have specific issues, including high costs and a shortage of qualified CMR-trained doctors.^4^ Furthermore, expert analysis requires significantly more time. A multicentre study by Bhuva et al.^7^ reported that specialist analysis of CMR takes 9–19 min. Compared with automated analysis, human analysis was 186 times slower (13 min vs. 0.07 min).^7^ Despite this, manual delineation by cardiologists or a semi-automatic approach remains the mainstay in current clinical practice.^8^

The integration of artificial intelligence (AI), particularly deep learning (DL), has transformed medical image interpretation by allowing models to learn hierarchical features directly from data rather than handcrafted descriptors.^9^ In most imaging applications, this is achieved with convolutional neural networks (CNNs), which apply learnable filters to detect edges, textures, and higher-level patterns, while pooling layers aggregate information across spatial scales.^10^ CNNs were first used for image-level classification, but were later adapted for dense, pixel-wise prediction through fully convolutional networks (FCNs) and encoder–decoder designs that replace the final fully connected layers with upsampling operations.^11,12^ In CMR, these architectures enable automatic delineation of cardiac structures, such as the left and right ventricles (LV and RV) and myocardium, facilitating the reproducible quantification of cardiac biomarkers, including ejection fraction, ventricular mass, and wall thickness.

Among CNN-based segmentation models, U-Net represents a domain-defining advancement specifically designed for biomedical segmentation. U-Net retains the convolutional backbone of CNNs but introduces a symmetric encoder–decoder structure with skip connections that fuse high-level contextual features with low-level spatial detail, allowing accurate segmentation even from relatively small annotated datasets.^13^ Subsequent innovations, including V-Net, DenseNet, and attention-based U-Nets, have extended this concept to three-dimensional (3D) volumes and multi-scale contexts, further improving performance in complex cardiac imaging tasks.^14^

Beyond static segmentation, cine CMR captures the heart's cyclical motion, requiring architectures capable of modelling temporal dynamics. Recurrent neural networks (RNNs), particularly those employing long short-term memory (LSTM) units, are well-suited for analysing sequential data. Their ability to propagate temporal dependencies enables motion-guided segmentation, thereby improving frame-to-frame consistency and functional quantification. For example, Qian et al.^15^ demonstrated joint motion estimation and segmentation of cine CMR using a recurrent U-Net, and Ghoul et al.^16^ reported multi-frame registration for automated ventricular function assessment in single-breath-hold cine MRI. While RNNs have been applied in niche contexts, such as motion artefact reduction, their primary value in CMR lies in leveraging temporal information to capture cardiac deformation and dynamics more comprehensively than slice-based CNNs.

Generative adversarial networks (GANs)^17^ and domain adaptation techniques^18^ have further advanced the field by enabling robust data augmentation, reducing domain gaps, and synthesizing realistic CMR images when annotated data are limited. These approaches are particularly valuable for multicentre applications, allowing greater generalisability by addressing cross-vendor and protocol variability.

This systematic review and meta-analysis aimed to critically evaluate the applications of DL in CMR imaging across three task domains:

- **Image segmentation:** pixel- or voxel-level prediction for delineating cardiac structures (e.g. LV, RV, myocardium) and deriving quantitative biomarkers, such as ventricular volumes, ejection fraction, and myocardial mass.
- **Diagnosis:** patient-level identification and differentiation between cardiac disease states or phenotypes (e.g. hypertrophic vs. dilated cardiomyopathy).
- **Prediction:** patient-level modelling of future outcomes, such as incident heart failure, arrhythmic events, or mortality.

The objectives of this review are as follows:

1. Provide pooled and comprehensive performance estimates of DL models across these CMR tasks.
2. Map architectural and training trends, including dimensionality (2D vs. 3D), network families (U-Net, nnU-Net, RNNs, transformers), and dataset provenance (public vs. non-public, single vs.. multi-vendor).
3. Assess reported evidence related to interpretability, calibration, and workflow integration readiness, and identify gaps limiting clinical translation.

## Methods

### Design

The protocol was registered in the International Prospective Register of Systematic Reviews (PROSPERO) under the registration number CRD42023439659, and the review adhered to the Preferred Reporting Items for Systematic Reviews and Meta-Analyses (PRISMA) guidelines.^19^ The PICO approach for this systematic review comprised: participants (*P*), both healthy individuals and patients with cardiac conditions, to investigate the application of DL in improving CMR; the intervention (I), DL-based applications; the control (C), conventional methods and standard approaches; and the outcomes (O), sensitivity and specificity analysis.

### Selection criteria

We included all studies that reported the clinical applications of DL in CMR, including image segmentation, prediction, and diagnosis. Furthermore, only studies published in English were considered. Medical imaging techniques presented in conference papers, review studies, and book chapters were excluded from this systematic review. Although there is substantial evidence of the use of AI in the literature, only studies focused on DL were considered. Studies on vascular MRI were excluded from the systematic review. Finally, animal studies were also excluded.

### Search strategy and data sources

A thorough search was conducted in various databases to identify studies that focused on different types of DL applications, including image segmentation, prediction, and diagnosis. The searched databases included MEDLINE, Embase, Scopus, and Web of Science. The keywords used in the search included DL OR artificial intelligence OR AI, cardiac OR heart, MRI OR magnetic resonance imaging, segmentation and prediction, and diagnosis. The search was conducted on 31 July 2025, and limited to studies published within the last five years (2020–2025).

### Data extraction

All search results were imported to the reference manager EndNot. Duplicate records were removed, and a combined research file was uploaded to Rayyan (Cambridge, MA: Rayyan Systems, Inc.), a software specifically built for conducting systematic reviews. Two reviewers (FA and MA) independently screened the titles and abstracts and applied the eligibility criteria. Any discrepancies were resolved through structured discussions with a third reviewer (AP) until a consensus was reached. Relevant information was extracted from each included study, including (1) the specific DL application employed (such as segmentation, prediction, etc.), (2) the sample size in terms of patients, images, and scans, (3) details about the neural network architecture and training process utilized, and (4) the accuracy assessment results reported in each study. Additionally, we recorded extra task-specific details relevant to the study objectives. For example, in the case of segmentation, the segmented area was extracted, and for prediction and diagnosis, comprehensive information regarding the investigated pathologies was extracted. In addition to technical performance, we extracted whether each study reported aspects of clinical translatability, including the use of interpretability methods (e.g. saliency maps, Grad-CAM), calibration or decision-curve analyses, and any information on workflow integration, runtime, or hardware requirements.

### Risk of bias assessment

Two reviewers (FA and MA) independently performed quality assessments using the Checklist for Artiﬁcial Intelligence in Medical Imaging (CLAIM)^20^ and Quality Assessment of Diagnostic Accuracy Studies-2 (QUADAS-2).^21^ The CLAIM is a checklist comprised of 42 items designed to assess AI research in medical imaging. Each item on the list was evaluated on a 2-point scale, with studies receiving a score of either 0 or 1 for each item. The scores for each item were summed to determine the overall CLAIM score. All items in the checklist carry the same weight. QUADAS-2 evaluates four key domains: (1) patient selection, (2) index test, (3) reference standard, and (4) flow and timing. Each domain was assessed for risk of bias. The assessments for both risk of bias were categorized as low, high, or of concern.

### Statistical analysis

All statistical analyses were conducted using MetaDisc Version 1.4, which was used to perform the meta-analysis of diagnostic accuracy and segmentation performance across the included studies. The analysis involved computing key diagnostic performance measures using true positive (TP), false positive (FP), true negative (TN), and false negative (FN) values with corresponding 95% confidence intervals (CI). These measures were calculated for each study and pooled to provide an overall summary of the diagnostic performance. In cases where one or more of the diagnostic parameters (TP, FP, TN, or FN) were not explicitly reported in the original studies, they were derived using the following standard diagnostic test formulae:

- **Sensitivity (Se):** TP/TP + FN, the sensitivity of the test for identifying actual positive cases
- **Specificity (Sp):** TN/TN + FP, quantifying the ability of the test to classify negatives correctly
- **Accuracy:** TP + TN/TP + TN + FP + FN, providing an overall measure of the test's correctness

Each of these measurements provides helpful information on the diagnostic precision of DL algorithms for segmentation and diagnosis based on CMR data. The following definitions hold for these vital diagnostic measurements:

- **True Positives (TP):** Those cases diagnosed correctly as positive (correctly diagnosing a patient with cardiac disease)
- **False Positives (FP):** Misclassifications as positive (a healthy patient being diagnosed with cardiac disease)
- **True Negatives (TN):** Those correctly diagnosed as negative (appropriately ruling out the disease from a healthy patient)
- **False Negatives (FN):** Cases incorrectly classified as negative (miss of cardiac disease in a patient with the disease)

When primary studies did not provide the complete 2×2 contingency table, we reconstructed missing TP, FP, TN, and FN values algebraically from the reported diagnostic metrics (e.g. sensitivity, specificity, accuracy) and the study sample size, assuming internal consistency of the published results. Where prevalence was not stated explicitly, we estimated it from the reported number of diseased and non-diseased cases in each cohort rather than imposing an external prior. The reconstructed counts were then treated as fixed inputs to the bivariate random-effects model; no additional multiple-imputation or probabilistic sampling was performed.

A bivariate random-effects model was employed to control for variation across studies, as it accounts for differences in study characteristics, such as network architecture, 2D vs. 3D imaging approaches, and dataset diversity. This model is appropriate, given the expected variability in diagnostic accuracy across studies involving different imaging techniques. By assuming that true effect sizes vary between studies, the random-effects model provides a more robust and generalizable pooled estimate of diagnostic accuracy. Statistical significance was determined at a *P*-value of less than 0.05, and heterogeneity was quantified using the I-squared (I^2^) statistic, with an I^2^ of 75% or above indicating high heterogeneity across studies. To facilitate better visualization of the pooled results, forest plots of sensitivity, specificity, and area under the curve (AUC) were produced, allowing for the graphical presentation of individual and combined results from the studies.

## Results

### Search and study selection

**Figure 1:**
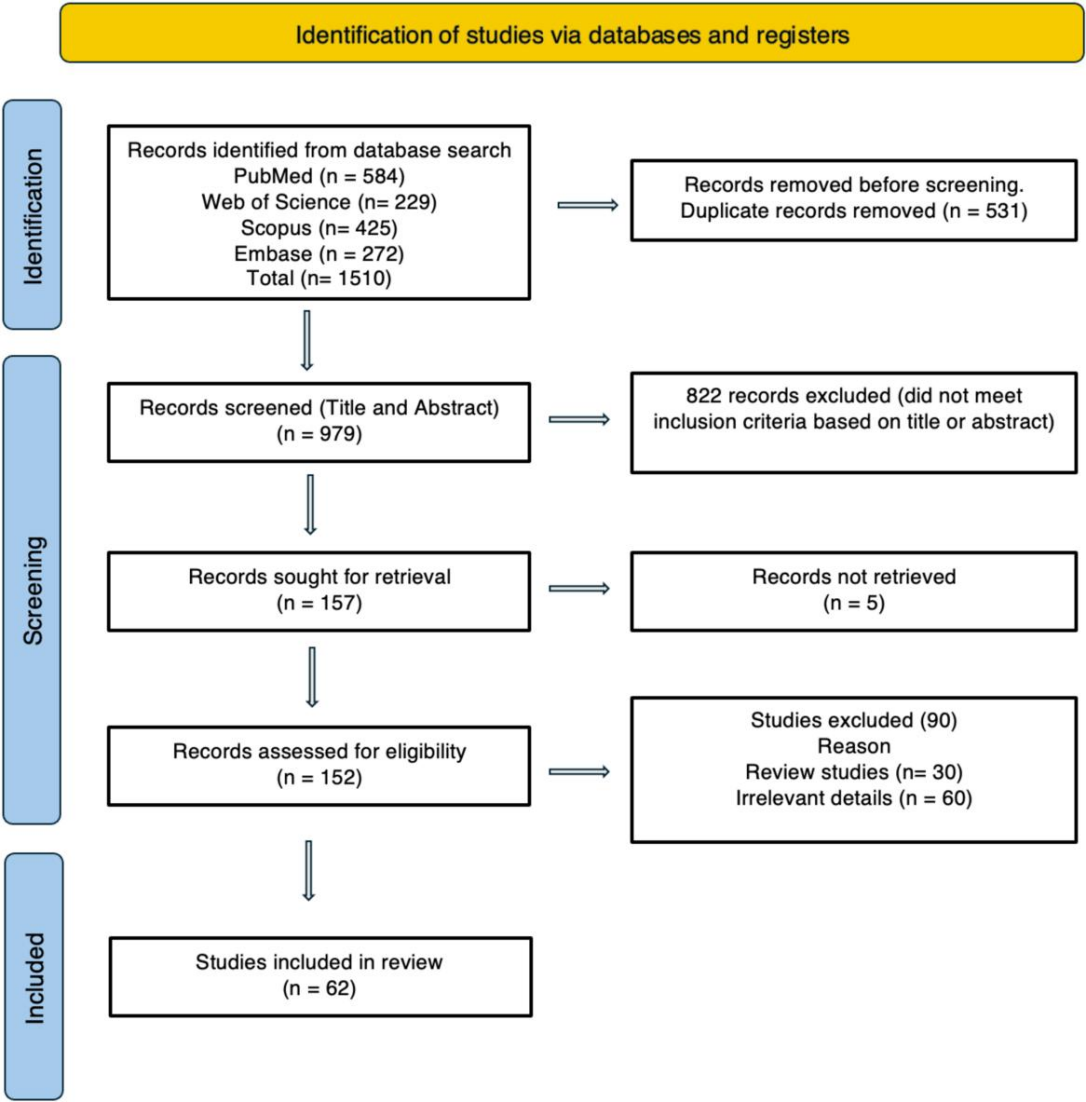
PRISMA flow diagram of the systematic review.

The initial search identified 1510 articles (PubMed: 584; Embase: 272; Web of Science: 229; and Scopus: 425). After removing 531 duplicate studies, 979 were screened. A total of 822 articles did not meet the inclusion criteria based on their titles and abstracts. After removing all non-relevant articles, 62 studies were included in the systematic review. *Figure 1* shows a PRISMA flow diagram.

### Characteristics of included studies

A total of 31 studies used data from subjects, whereas the remaining studies used a mixture of public and private datasets. Public datasets commonly used included the Automated Cardiac Diagnosis Challenge (ACDC), Multi-Centre, Multi-Vendor & Multi-Disease Cardiac Image Segmentation (M&Ms), MRPEAT, and the Sunnybrook Cardiac Data. *Table 1* provides an overview of the major publicly available CMR datasets identified across the included studies, including their sample size, vendor diversity, primary imaging sequences, and clinical focus. Other studies used single-centre institutional datasets or multicentre datasets that were not publicly released. Most studies used supervised training (91.94%) over semi-supervised training. Most studies used the U-Net network architecture (70.97%, *n* & 44).

**Table 1:** Publicly available CMR datasets used across the included studies

DATASET	VENDOR DIVERSITY	SAMPLE SIZE	CMR SEQUENCES	PRIMARY FOCUS
*ACDC*	Multi-vendor	150 subjects	CINE MRI	LV/RV/MYO segmentation
*M&MS*	Multi-vendor (6 centres)	320 subjects	CINE MRI	Multi-vendor generalization, LV/RV segmentation
*MRPEAT*	Multi-centre	∼200 subjects	Cine MRI + complementary sequences	segmentation and reconstruction
*SUNNYBROOK CARDIAC DATA (LVSC)*	Single vendor (Siemens)	45 subjects	Cine MRI ± LGE	LV segmentation, myocardial pathology assessment
*UK BIOBANK CMR*	Nationwide, multi-scanner	∼45 000 subjects	Cine MRI + T1/T2 mapping + tagging	Population-level LV/RV segmentation and phenotype extraction

A total of 45 studies were related to the image segmentation. Supplementary data online, *Table S1* shows the details of studies that focused on image segmentation. The findings of the included studies consistently demonstrated that DL models are effective for LV and RV segmentation. Furthermore, they reported that the performance levels were comparable to or surpassed those of expert manual annotations. Some studies have highlighted specific benefits, such as the robustness of DL methods in handling outlier cases.^22^ Additionally, the integration of innovative techniques, such as bidirectional convolutional LSTM, late fusion multi-encoder structures, and semi-supervised learning approaches, contributed to reducing segmentation failures.

Of the 62 studies included in this systematic review, 9 focused on diagnosis and 8 on prediction. All studies related to diagnosis and prediction used supervised training. The findings showed that the DL models consistently showed improved accuracy in diagnosing and predicting outcomes (see Supplementary data online, *Table S2*). DL approaches have proven effective in predicting outcomes and diagnosing conditions, such as heart failure with reduced ejection fraction (HFrEF), left ventricular hypertrophy (LVH), and cardiac amyloidosis.

### Meta-analysis

#### Prediction and diagnosis

**Figure 2:**
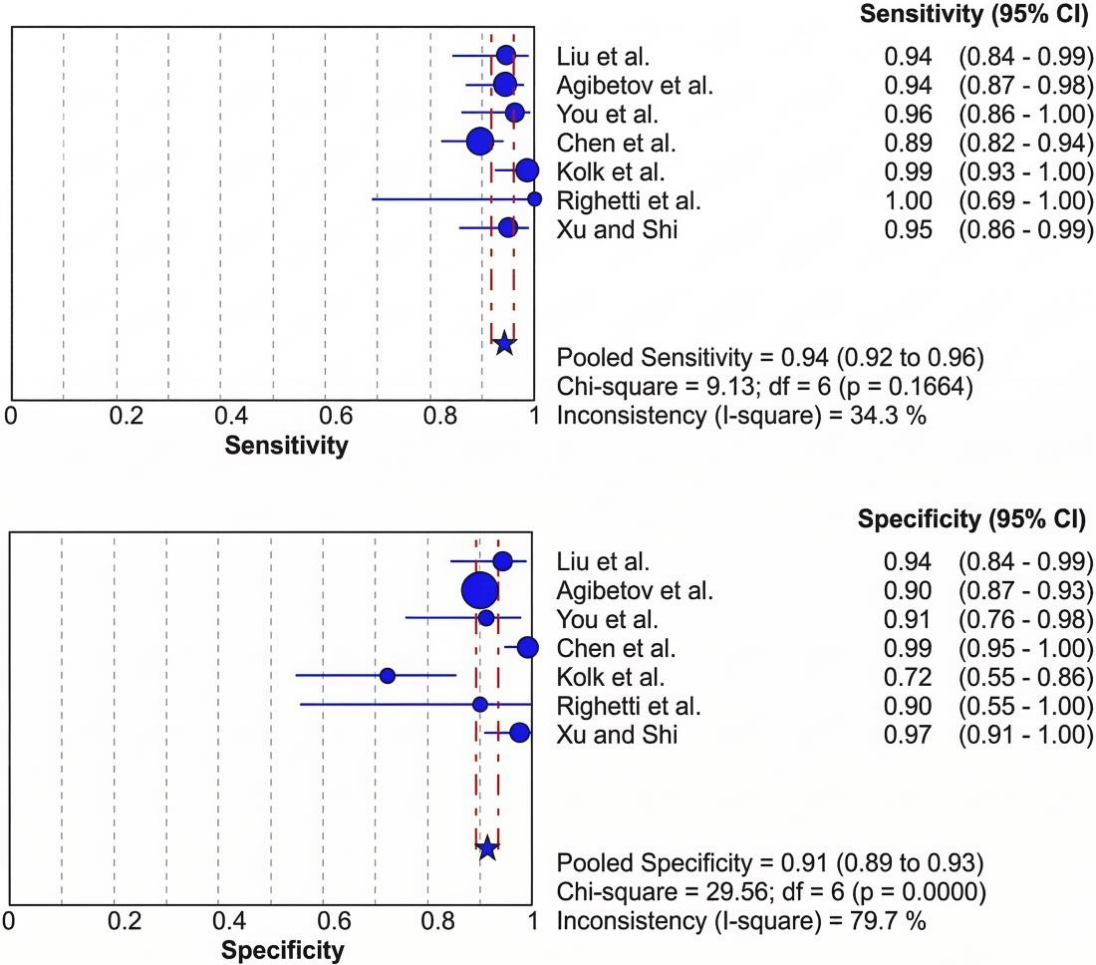
Forest plots of pooled sensitivity and specificity for deep learning models in cardiac MRI diagnosis and prediction.

A meta-analysis was conducted to determine the diagnostic accuracy of CMR using DL models, pooling data from seven studies. The pooled sensitivity was 0.94 (95% CI: 0.92–0.96), with low heterogeneity (I^2^ & 34.3%, *P* & 0.1664), indicating a high and consistent ability of DL models to correctly identify actual positive cases. The pooled specificity was 0.91 (95% CI: 0.89–0.93) with substantial heterogeneity (I^2^ & 79.7%, *P* < 0.001) (*Figure 2*), suggesting variability in the correct identification of true negatives across studies. The symmetric summary receiver operating characteristic (SROC) analysis yielded an AUC of 0.9831 (SE = 0.0044) (see Supplementary data online, *Figure S1*), reflecting an excellent overall diagnostic performance. The Q* index was 0.9432 (SE & 0.0088), confirming a balanced sensitivity and specificity at the optimal decision threshold. These results demonstrate that DL-based CMR interpretation achieves near-expert diagnostic accuracy, particularly for disease detection and prediction tasks.

#### Segmentation

**Figure 3:**
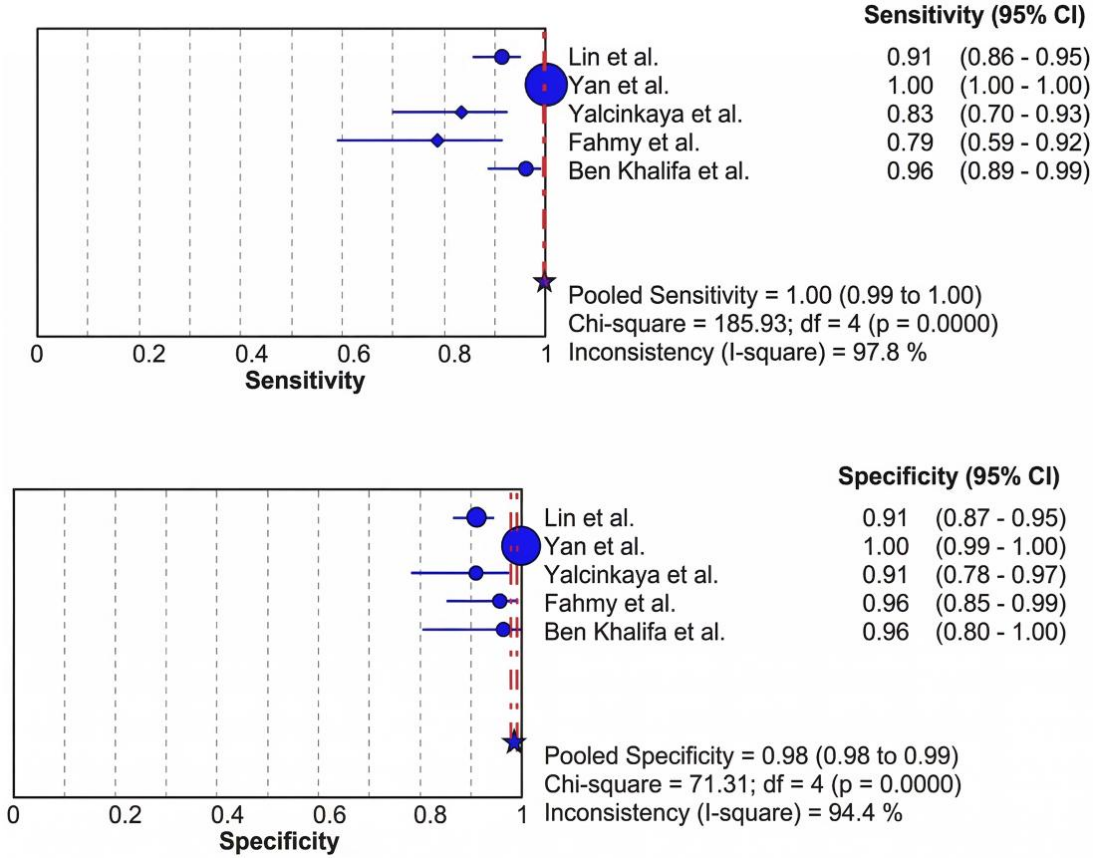
Forest plots of pooled sensitivity and specificity for deep learning models in cardiac MRI segmentation.

A meta-analysis was performed to evaluate the segmentation performance of DL models in CMR by pooling data from five studies. The pooled sensitivity was 1.00 (95% CI: 0.99–1.00), indicating a statistically significant and near-perfect ability of DL-based segmentation to correctly identify target cardiac structures. The pooled specificity was 0.98 (95% CI: 0.98–0.99) (*Figure 3*), reflecting a statistically significant accuracy in correctly excluding non-target regions. Despite these high-performance metrics, heterogeneity was substantial for both sensitivity (I^2^ = 97.8%, *P* < 0.001) and specificity (I^2^ = 94.4%, *P* < 0.001), suggesting considerable variability between studies. The SROC analysis demonstrated excellent and statistically significant discriminatory ability, with an AUC of 0.9940 (SE = 0.0051) (see Supplementary data online, *Figure S2*) and a Q* index of 0.9692 (SE & 0.0154). These findings confirm that DL models deliver highly accurate and statistically robust segmentation in CMR. Although the pooled sensitivity approached unity, this value should not be interpreted as evidence of uniformly perfect segmentation performance. It reflects aggregation across highly heterogeneous studies that differ in network architecture, anatomical targets, evaluation metrics, and dataset provenance, and therefore represents an average across diverse experimental conditions rather than a clinically error-free benchmark.

### Quality assessment

**Figure 4:**
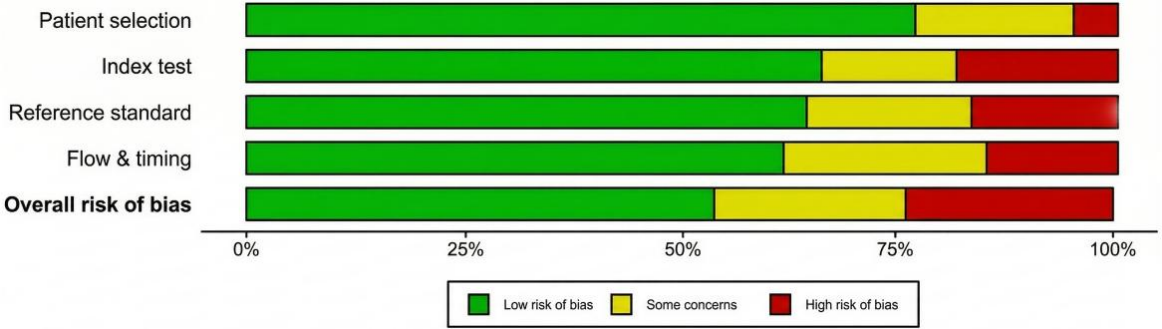
Summary plot of risk of bias.

We assessed the quality of the included studies using the QUADAS-2 tool across four domains: patient selection, index test, reference standard, and flow and timing. Overall, 36 studies (58.06%) were rated as having a low risk of bias, 14 (22.58%) had some concerns, and 12 (19.35%) were rated as having a high risk of bias (see supplementary data online, *Figure S3*, and *Figure 4*). Most studies employed appropriate patient selection and reliable reference standards, although some lacked clarity regarding blinding procedures and timing between tests. The average CLAIM score for all studies was 86.41%. The highest CLAIM score was assessed in Penso et al.^23^ (97.62%), whereas the lowest score was evaluated in Das et al.^24^ (71.43%) (*Table 2*)

**Table 2:** CLAIM checklist score

Author	Title/Abstract (n/2)	Introduction (n/2)	Methods (n/28)	Results (n/5)	Discussion (n/2)	Other information (n/3)	Total Score (n/42)	% Score
*Parikh et al.*	2	1	22	3	1	2	31	73.81
*Khalil et al.^22^*	1	2	25	4	2	3	37	88.10
*Hu et al.^2^*	2	2	25	3	2	3	37	88.10
*Wang et al.^4^*	2	2	23	4	1	3	35	83.33
*Gao et al.^25^*	2	2	22	5	2	3	36	85.71
*Ribeiro et al^26^*	1	2	25	3	2	2	35	83.33
*Gavirni et al.*	2	2	26	3	1	2	36	85.71
*Diao et al.^27^*	2	2	25	4	2	3	38	90.48
*Das et al.^24^*	1	1	22	3	0	3	30	71.43
*Chen et al.^28^*	1	2	25	3	2	2	35	83.33
*Wang et al.^4^*	1	2	24	2	1	3	33	78.57
*Chen et al.^28^*	1	2	23	3	2	3	34	80.95
*Akesson et al.*	1	2	24	3	2	3	35	83.33
*Ammann et al.*	2	2	25	4	2	3	38	90.48
*Yan et al.^29^*	2	2	23	4	2	3	36	85.71
*Chang et al.*	2	2	24	5	2	3	38	90.48
*Penso et al.^23^*	2	2	26	4	2	3	39	92.86
*Wang et al.^4^*	2	2	25	4	1	2	36	85.71
*Lin et al.^30^*	1	2	27	4	1	2	37	88.10
*Arai et al.*	2	2	27	4	1	2	38	90.48
*Agibetov er al.^31^*	2	2	27	4	2	3	40	95.24
*Popescu et al.^32^*	2	2	25	4	2	3	38	90.48
*Yalcinkaa et al.*	1	2	26	4	1	2	36	85.71
*Wang and Zhang*	1	2	24	4	1	3	35	83.33
*Xue et al.^3^*	2	2	24	4	2	2	36	85.71
*Fahmy et al.*	2	2	27	5	2	2	40	95.24
*Vesal et al.^33^*	1	2	25	5	1	2	36	85.71
*Wang et al.^4^*	2	2	24	4	2	3	37	88.10
*You et al.*	2	2	25	4	1	3	37	88.10
*Song et al.*	1	2	24	4	1	3	35	83.33
*Ammar et al.*	1	2	25	4	1	2	35	83.33
*Zarvani et al.^34^*	2	2	24	4	1	2	35	83.33
*Galea et al.*	1	2	25	4	2	3	37	88.10
*Penso et al.^23^*	2	2	28	4	2	3	41	97.62
*Bartoli et al.^35^*	2	2	27	5	2	2	40	95.24
*Vesal et al.^33^*	1	2	27	4	1	3	38	90.48
*Graves et al.*	1	2	23	3	1	2	32	76.19
*Abdeltawb et al.*	1	2	25	3	1	3	35	83.33
*Tran et al.*	1	2	25	4	1	2	35	83.33
*Diller et al.*	2	2	26	4	2	3	39	92.86
*Qin et al.*	2	2	25	4	2	2	37	88.10
*Zhao et al.^36^*	2	2	25	4	1	2	36	85.71
*Luo et al.*	1	2	25	4	1	2	35	83.33
*Du et al.^37^*	2	2	25	4	1	2	36	85.71
*Liu et al.*	1	2	26	4	1	2	36	85.71
*Alskaf et al.^38^*	2	2	24	4	2	2	36	85.71
*Alskaf et al.^38^*	2	2	25	4	2	2	37	88.10
*Amyar et al.^39^*	1	1	23	3	1	1	30	71.43
*Baraboo et al.^40^*	2	2	26	4	1	2	37	88.10
*Barón et al.*	1	2	25	3	0	3	34	80.95
*Ben Khalifa et al.^41^*	2	2	27	4	1	2	38	90.48
*Chen et al.^28^*	2	2	26	4	2	3	39	92.86
*Cockrum et al.*	2	2	27	5	2	2	40	95.24
*Elizar et al.*	2	2	25	3	1	2	35	83.33
*Hatfaludi et al.*	2	2	26	4	1	2	37	88.10
*Kim et al.^42^*	2	2	24	4	1	1	34	80.95
*Kolk et al.^43^*	2	2	25	4	1	2	36	85.71
*Righetti et al.^44^*	2	2	26	3	2	2	37	88.10
*Shaaf et al.*	2	2	27	3	1	1	36	85.71
*Xu and Shi^45^*	2	2	25	3	1	2	35	83.33
*Leite et al.^46^*	2	2	26	4	2	3	39	92.86
*Pham et al.*	1	2	27	4	2	3	39	92.86

Across the included studies, reporting of clinical-translatability features was limited. Only a small minority explicitly presented interpretability outputs (e.g. saliency maps or Grad-CAM), and calibration analysis or decision-curve analysis was rarely performed. Information on workflow integration, inference time, or hardware requirements was inconsistently provided and could not be synthesized quantitatively.

### Segmentation metrics

**Figure 5:**
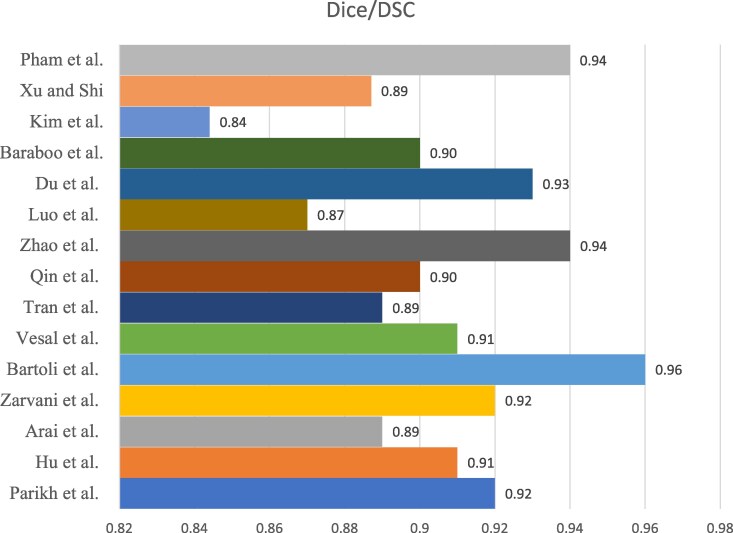
Dice/DSC score values reported in studies.

Fifteen studies reported the Dice similarity coefficient (DSC), most of which employed a UNet-based architecture. The pooled mean DSC was 0.91 ± 0.03, indicating the strong and consistent performance of the DL models in image segmentation (*Table 3*, *Figure 5*). The highest DSC value was reported by Bartoli et al.^35^ (0.96), reflecting a near-perfect overlap with the reference segmentations. Six studies reported HD, with a pooled mean of 8.99 ± 6.45 mm. The HD values varied substantially across studies, partly reflecting the differences in segmentation dimensionality. 2D segmentation studies tend to report lower HD values, as seen in Hu et al.^2^ (4.78 mm), which suggests highly precise boundary alignment. In contrast, studies using 3D segmentation, such as Vesal et al.^33^ reported higher HD values (20.30 mm), consistent with the known sensitivity of 3D HD to outlier boundary deviations and greater through-plane voxel spacing. These methodological differences should be considered when interpreting the pooled HD results (*Table 4*).

**Table 3:** Dice/DSC score values reported in studies

SR	AUTHOR	NETWORK ARCHITECTURE	DICE/DSC
*1*	Parikh et al.^47^	Dense Unet with bidirectional convolution LSTM	0.92
*2*	Hu et al.^2^	U-net	0.91
*3*	Arai et al.	U-Net	0.89
*4*	Zarvani et al.^34^	Residual Network of Residual Network, ROR-Unet	0.92
*5*	Bartoli et al.^35^	DenseNet architecture	0.96
*6*	Vesal et al.^33^	A 3D dilated residual U-Net (3D DR-UNet)	0.91
*7*	Tran et al.	U-Net	0.89
*8*	Qin et al.	Dense V-Net	0.90
*9*	Zhao et al.^36^	U-Net	0.94
*10*	Luo et al.	U-Net	0.87
*11*	Du et al.^37^	Multi-scale context-aware module	0.93
*12*	Baraboo et al.^40^	U-net	0.9
*13*	Kim et al.^42^	U-net	0.844
*14*	Xu and Shi^45^	U-net	0.887
*15*	Pham et al.	CapNet	0.9399

**Table 4:** HD values reported in the studies

SR	AUTHOR	OVERALL
*1*	Hu et al.^2^	4.78
*2*	Vesal et al.^33^	20.30
*3*	Tran et al.	5.1
*4*	Zhao et al.^36^	7.5
*5*	Du et al.^37^	11.8
*6*	Xu and Shi^45^	4.48

### Diagnostic and prediction metrics

*Table 5* presents the collected data on sensitivity, specificity, accuracy, and precision from studies that evaluated the performance of various DL models. Fourteen studies reported sensitivity. The highest sensitivity was reported by Righetti et al.^44^ (100%), suggesting an exceptional ability to detect actual positive cases. Fourteen studies reported accuracy. The highest accuracy of 100% was reported by Righetti et al.^44^ indicating a very high overall performance in correctly classifying both positive and negative cases. Specificity, which measures the ability to correctly identify negative cases, was reported in seven studies. Yan et al.^29^ achieved a specificity of 99.8%, which reflects a strong performance in avoiding false positives. Precision was reported in five studies, with the highest being 97%, as demonstrated by Ben Khalifa et al.^41^ which showed the model's effectiveness in accurately identifying positive cases out of all instances flagged as positive.

**Table 5:** Studies reporting accuracy, sensitivity, specificity, and precision

AUTHOR	CLINICAL APPLICATION	ACCURACY (%)	SENSITIVITY (%)	SPECIFICITY (%)	PRECISION (%)
*YAN ET AL.* ^29^	Segmentation	99.95	99.97	99.86	—
*LIN ET AL.* ^30^	Segmentation	99.11 (0.33)	91.24 (2.62)	—	90.61 (2.85)
*YALCINKAYA ET AL.*	Segmentation	—	82.6	91.8	—
*FAHMY ET AL.*	Segmentation	89	77	96	—
*PENSO ET AL.* ^23^	Segmentation	—	93.5 (1.1)	—	—
*LIU ET AL.*	Segmentation	—	94	—	—
*AGIBETOV ET AL.* ^31^	Diagnosis	—	94	90	—
*YOU ET AL.*	Diagnosis	96.79	95.24	—	—
*ALSKAF ET AL.* ^38^	Diagnosis	81	—	—	—
*ALSKAF ET AL.*	Diagnosis	75	—	—	—
*BARÓN ET AL.*	Diagnosis	89.6	—	—	90.7
*CHEN ET AL.* ^28^	Diagnosis	92.4	89.5	99.2	—
*KOLK ET AL.* ^43^	Prediction	84.4	98.1	72.6	—
*RIGHETTI ET AL.* ^44^	Prediction	100	100	90	—
*SHAAF ET AL.*	Prediction	91	95	—	94
*XU AND SHI* ^45^	Prediction	96.3	94	—	96

## Discussion

### Findings of the review

Recent advances in DL have markedly improved the accuracy and efficiency of CMR segmentation and diagnostic tasks; however, the comparative performance of different architectures, training strategies, and dataset types shows substantial variability. Notably, advanced architectures like Dense U-Net with bidirectional convolutional LSTM achieved DSC of 0.92 across the ACDC and M&Ms datasets, delivering both high accuracy and strong generalisability to heterogeneous, multi-institutional populations.^47^ Likewise, DenseNet by Bartoli et al.^35^ outperformed other architectures in challenging segmentation tasks, achieving DSC of 0.96 for complex trabeculation analysis, while Residual Network of Residual Networks by Zarvani et al.^34^ also performed well and achieved strong LV boundary segmentation (DSC 0.92).

Despite the dominance of U-Net variants, their DSC demonstrated a considerable range from 0.84 to 0.94 (Kim et al.^42^ Zhao et al.^36^) reflecting sensitivity to anatomical target, dataset composition, and centre provenance. Novel or automated strategies, such as the multi-encoder nnU-Net by Khalil et al.^22^ and triple-stage U-Net by Chen et al.^28^ have shown greater robustness, particularly in outlier and multi-centre cases. Furthermore, a shift to 3D architectures, for example, Vesal et al. and their 3D DR-UNet^33^ improved volumetric representation (DSC 0.91) at the cost of increased HD up to 20.3 mm, highlighting persistent boundary errors from through-plane misalignments. In contrast, 2D approaches remained more reliable for well-defined structures (Hu et al., DSC 0.91; HD 4.78 mm.^2^)

The provenance of the dataset substantially influenced the real-world performance. Models trained and evaluated on public, multi-vendor datasets such as ACDC, M&Ms, and Sunnybrook, such as Parikh et al.^47^ Khalil et al.^22^ Ribeiro et al.^26^ Penso et al.^23^ consistently yielded more generalizable models than those developed on single-centre and private-only studies (Kim et al.^42^ Baraboo et al.^40^) reinforcing the need for open benchmarking to minimize overfitting and improve clinical translation.

In diagnostic and prognostic applications, R Fast CNN by Gao et al.^25^ Res-UNet by Diao et al.^27^ and hybrid rVAE models by Kolk et al.^43^ all achieved high sensitivity and AUC for disease classification and outcome prediction, particularly when clinical data were integrated with CMR images. Multimodal and spatiotemporal residual approaches further demonstrated the value of domain fusion for detecting subtle pathologies,^39^ although robust external and prospective validation remains insufficient.

#### Heterogeneity analysis

Structured analysis of post-hoc heterogeneity explicitly links the high I^2^ values observed in the meta-analyses to the variability in architecture, segmentation dimensionality, dataset diversity, and vendor protocols. For example, 3D networks (Vesal et al., HD 20.3 mm)^33^ were more prone to through-plane errors than robust 2D approaches (Hu et al., HD 4.78 mm).^2^ Also, public, multi-vendor datasets (Khalil et al.^22^ Parikh et al.^47^) provided more reproducible estimates than single-vendor single-centre series, which often overestimated accuracy due to overfitting. Segmentation difficulty also varied by anatomical structure, with LV segmentation consistently exceeding RV and atrial segmentation (Popescu et al.^32^ Zhao et al.^36^) Tasks involving LGE images typically achieved lower agreement than cine MRI, reflecting the added complexity of scar and tissue characterization. In addition, the included studies differed in whether they addressed single-class segmentation (e.g. LV cavity only) or multi-class segmentation of several cardiac structures, such as LV, RV, and myocardium. Single-class LV segmentation studies, including those by Bartoli et al. (DSC 0.96)^35^ and Zarvani et al. (DSC 0.92),^34^ generally reported higher DSC than multi-class applications like Kim et al.^42^ where U-Net-based segmentation of multiple tissues achieved lower mean DSC (0.844). Dataset size also varied markedly between studies, ranging from small single-centre cohorts with only tens of patients to large public or multi-centre datasets such as ACDC, M&Ms, MRPEAT and UK Biobank. Smaller, single-centre datasets (e.g. Chen et al.^28^ Lin et al.^30^ Zhao et al.^36^, Du et al.^37^) tended to show greater variance and a higher risk of overfitting than larger multi-centre studies based on ACDC, M&Ms and UK Biobank (e.g. Parikh et al.^47^ Khalil et al.^22^ Hu et al.^2^) and vendor-specific protocols and artefact patterns further amplified these performance differences, underscoring the need for routine benchmarking on multicentre, multi-sequence data when interpreting pooled summary estimates and drawing generalizable conclusions. The near-perfect pooled segmentation sensitivity must be interpreted cautiously in the context of very high heterogeneity. Rather than indicating consistently flawless performance, it reflects aggregation across studies that vary widely in dataset size, anatomical focus, evaluation protocols, and reference standards. In particular, pooled results blend 2D and 3D models, public multi-vendor datasets, and single-centre series, and tasks ranging from LV-only to multi-class segmentation, all of which showed materially different Dice and HD values.

### Interpretability and clinical adoption

Although DSC is the most common metric for segmentation validation, high scores may obscure clinically critical errors, as these indices are less sensitive to localized segmentation failures in thin myocardium or trabeculated regions. Meanwhile, HD provided additional sensitivity to boundary error, with higher HD observed for 3D models (Vesal et al., HD 20.3 mm^33^) and more consistency in 2D networks (Hu et al., 4.78 mm,2 Xu and Shi, 4.48 mm.^45^) For diagnostic and prognostic models, AUC, sensitivity, and specificity were commonly reported (Kolk et al.^43^ Diao et al.^27^ and Agibetov et al.^31^) but calibration and risk stratification utility were rarely assessed, limiting their clinical applicability.

Interpretability and explainable AI (XAI) are similarly under-addressed. While methods such as Grad-CAM or saliency mapping are recommended,^48^ they remain rare among published CMR DL studies. The work of Xu and Shi,^45^ which combined DL with radiomics, is a notable exception. Broader adoption of XAI frameworks will be essential to improve transparency, clinician trust, and regulatory acceptability. Consequently, while we set out to assess interpretability, calibration, and workflow readiness, the available data allowed only a narrative synthesis rather than formal quantitative analysis, highlighting an important evidence gap for clinical translation.

### CMR-specific comparison and workflow barriers

This review, focused solely on CMR, highlights specific challenges and advances compared to prior cross-specialty or multi-modality AI reviews, such as Aggarwal et al.^49^ and Alskaf et al.^38^ Across datasets, LV segmentation is consistently more accurate than RV and atrial or scar segmentation, reflecting both anatomical and imaging differences. Public, multi-vendor datasets such as ACDC and M&Ms underpin robust cross-site generalization, while homogeneous, single-centre, and private data may artificially inflate outcomes and do not address workflow or domain shift barriers. Sequence diversity between cine and LGE images further complicates standardization, calibration, workflow integration, and open annotation provenance are rarely considered in single-site studies. These issues form a translational barrier that only larger, harmonized, and prospectively validated pipelines can address.

Reporting of computational cost and inference speed was sparse. Where stated, segmentation pipelines typically reported per-scan runtimes of seconds to a few minutes on contemporary GPUs, which would be compatible with offline clinical post-processing, but most studies did not specify hardware details or latency. Diagnostic and prognostic models rarely provided explicit throughput benchmarks, making it difficult to assess their suitability for real-time decision support. Standardized reporting of inference time, hardware configuration, and potential for CPU-only deployment would substantially improve evaluation of clinical feasibility.

### Limitations

Variability in dataset origin, imaging protocol, and reporting practices contributed to substantial heterogeneity in reported outcomes. Public challenge datasets provide standardized evaluation but may not reflect real-world variability, while single-centre private datasets risk overfitting to local imaging characteristics. Additionally, the high heterogeneity (97.8%) observed across studies can be attributed to differences in network architecture, such as the use of U-Net, ResNet, and transformer-based models, each with distinct capabilities and impact on model performance. Furthermore, the comparison of 2D vs. 3D imaging approaches introduced additional variability, as 3D models tend to capture more context and spatial relationships compared to 2D models, which may explain discrepancies in segmentation accuracy across studies. The diversity in datasets, including the variation in disease composition and data acquisition protocols, further contributed to this heterogeneity.

Some studies have inadequate reporting of their methods, such as patient demographics, results, or conclusions, which limits the ability to generalize the findings. Additionally, the lack of prospective, workflow-integrated trials is a major limitation, with most evaluations confined to post-hoc testing on historical data.

For several diagnostic studies, we reconstructed missing elements of the 2×2 tables from published summary metrics and cohort sizes, and estimated prevalence from the reported numbers of diseased and non-diseased participants. This approach assumes internally consistent reporting and does not propagate additional uncertainty from the reconstruction step, so the confidence intervals around pooled diagnostic accuracy may be mildly over-optimistic.

An additional concern is the domain gap between the datasets used in most studies and the full spectrum of cases encountered in routine practice. The majority of segmentation and diagnostic pipelines were trained and evaluated on relatively curated images with limited representation of severe motion artefacts, low contrast, implanted devices, or rare congenital and acquired pathologies. Only a few studies sought to address robustness explicitly, for example, by using GAN-based augmentation or late-fusion strategies to mitigate failures in outlier anatomies or across vendors. Systematic out-of-distribution testing, adversarial validation and explicit outlier-detection frameworks were seldom reported, leaving important questions about how these models perform under degraded image quality or previously unseen conditions.

The evaluation of DL models in the included studies was limited to few metrics, such as accuracy, DSC score, and HD, without considering other necessary measures, including recall and precision. Several studies were deemed to have a high risk of bias, particularly in the domains of reference standard and index test. For example, unblinded reference standard assessments, where the same annotators were involved in both creating the ground truth and evaluating the model output, could inflate reported performance by introducing confirmation bias. Similarly, unblinded index test results, where evaluators were aware of the model's predictions, might lead to more lenient acceptance of borderline segmentation errors. Selection bias was also present in some studies, for instance, through the inclusion of only high-quality images or specific patient subgroups. Such selective sampling can limit generalisability and result in overly optimistic performance metrics that may not hold in real-world settings where image quality and patient anatomy are more variable.

Additionally, the models used in the studies may have technical limitations, such as underfitting or overfitting, which can impact their performance. Most studies focused on evaluating DL model performance on CMR segmentation tasks without necessarily assessing their clinical utility or impact on patient outcomes. Furthermore, there may be publication bias towards studies reporting positive results, which could contribute to an overestimation of the effectiveness of DL models for CMR segmentation.

Due to the insufficient number of studies in certain subgroups and the lack of relevant data for subgrouping, we were unable to perform subgroup analyses that might have provided a deeper understanding of the sources of variation across studies.

### Research gaps and future directions

Despite growing interest in temporal modelling and multi-sequence integration, relatively few studies employ LSTM/RNNs for cine CMR (Qian et al.^15^ Wang & Zhang^50^) or fully domain-adaptive, multi-sequence pipelines (Penso et al.^23^ Khalil et al.^22^ Leite et al.^46^). Automated pipelines such as nnU-Net by Khalil et al.^22^ represent a promising step towards standardized, reproducible pipelines. However, this review did not identify work that systematically applies foundation models (e.g. SAM, vision transformers) or large language models for generalist, multimodal CMR analysis. Although foundation models have shown strong performance in natural-image segmentation, their direct applicability to CMR is currently limited. Models pretrained on non-medical images often underperform on CMR without domain adaptation, typically requiring fine-tuning on labelled cardiac datasets, prompt engineering or pretraining on large-scale medical imaging corpora to achieve clinically acceptable accuracy.^51–53^ Early work in medical imaging has also highlighted concerns about data-hungry training, domain shift, and hallucinated structure when such models are applied without careful adaptation and validation.^51–53^ These limitations mean that, at present, foundation models should be viewed as promising research tools rather than ready-to-deploy solutions in routine CMR practice.

Future research should prioritize rigorous multi-centre and prospective validation, explicit domain adaptation and sequence harmonization strategies, routine use of interpretability methods, and robust assessment of calibration and net clinical benefit. Addressing these gaps will be crucial for translating promising technical performance into reliable, clinically integrated tools.

## Conclusion

DL models in CMR now often match or exceed expert performance on technical benchmarks, but their clinical impact remains constrained by limited interpretability, a lack of calibration and prospective validation, and sparse benchmarking across representative, diverse populations. Overcoming these barriers will require methodologically rigorous, multi-centre studies and an emphasis on explainability, harmonization, and clinical utility rather than purely technical accuracy.

## Supplementary Material

qyag045_Supplementary_Data

## Data Availability

No new data were generated or analysed in support of this research.

## Lead author biography

Fatemah Aladwani is a PhD student in Medicine at the University of Dundee, United Kingdom. She holds a bachelor's degree in radiologic technology from Kuwait University and a master's degree with distinction in Medical Imaging from the University of Dundee. She has over a decade of clinical experience as a senior radiologic technologist at the Ministry of Health in Kuwait, specializing in MRI. Her research focuses on cardiac MRI and the application of AI to improve disease prediction. Her professional and academic journey is driven by a commitment to advancing diagnostic imaging and improving patient care outcomes.
