# Supplementary material for: Deep learning for cardiac MRI: performance evidence and barriers to clinical integration. A Systematic Review and Meta-Analysis
Source: Eur Heart J Imaging Methods Pract. 2026 Mar 16;4(1):qyag045. doi: 10.1093/ehjimp/qyag045 (PMC13007597; doi:10.1093/ehjimp/qyag045)
Supplement: qyag045_Supplementary_Data [file qyag045_supplementary_data.zip › Search Strategy SR.docx]

**Search Strategy Medline (Ebsco interface) July 2023**

|  | Deep learning [MeSH] |
| --- | --- |
|  | Deep learning |
|  | Artificial Intelligence [MeSH] |
|  | Artifical intelligence |
|  | AI |
|  | or/1-5 |
|  | Cardiac Imaging Techniques [MeSH] |
|  | Cardiac Imag* |
|  | or/7-8 |
|  | Magnetic Resonance Imaging [MeSH] |
|  | Magnetic Resonance Imaging |
|  | MRI OR CMR |
|  | or/10-12 |
|  | 6 and 9 and 13 |

**EMBASE (Ovid interface)**

|  | Deep learning/ |
| --- | --- |
|  | Deep learning.ti,ab. |
|  | Artificial Intelligence/ |
|  | Artifical intelligence.ti,ab. |
|  | AI.ti,ab. |
|  | or/1-5 |
|  | Cardiac Imaging/ |
|  | Cardiac Imag*.ti,ab. |
|  | or/7-8 |
|  | Nuclear Magnetic Resonance Imaging/ |
|  | Magnetic Resonance Imaging.ti,ab. |
|  | (MRI OR CMR).ti,ab. |
|  | or/10-12 |
|  | 6 and 9 and 13 |

**SCOPUS**

(“Deep learning” OR “Artifical Intelligence” OR AI) AND “Cardiac Imag*” AND (“Magnetic Resonance Imaging” OR MRI OR CMR)

**WEB OF SCIENCE**

(“Deep learning” OR “Artifical Intelligence” OR AI) AND “Cardiac Imag*” AND (“Magnetic Resonance Imaging” OR MRI OR CMR)

Databases search ended on July 2025.
