# Supplementary material for: Deep learning for cardiac MRI: performance evidence and barriers to clinical integration. A Systematic Review and Meta-Analysis
Source: Eur Heart J Imaging Methods Pract. 2026 Mar 16;4(1):qyag045. doi: 10.1093/ehjimp/qyag045 (PMC13007597; doi:10.1093/ehjimp/qyag045)
Supplement: qyag045_Supplementary_Data [file qyag045_supplementary_data.zip › Supplematray figure 3.pdf]

Study	D1	D2	D3	D4	Overall
Abdeltawab 2020	+	+	+	+	+
Agibetov 2021	+	+	+	+	+
Akesson 2023	+	X	X	-	X
Ammann et al. 2023	+	+	+	+	+
Ammar 2021	+	X	X	X	X
Arai 2022	+	+	+	+	+
Bartoli 2020	+	+	+	+	+
Chang 2022	+	+	+	+	+
Chen 2023	+	+	+	+	+
Das 2023	-	-	X	X	X
Diao 2023	+	+	+	+	+
Diller 2020	+	-	-	-	-
Du 2020	+	X	X	X	X
Fahmy 2021	+	+	+	+	+
Galea 2021	+	+	+	+	+
Gao 2023	+	+	+	+	+
Gavirni 2023	+	-	-	-	-
Graves 2020	+	-	-	-	-
Hu 2023	-	+	-	-	-
Khalil 2023	+	+	+	+	+
Lijia Wang 2023	+	-	-	+	-
Lin 2022	+	+	+	+	+
Liu 2020	+	X	X	X	X
Luo 2020	+	+	+	+	+
Parikh 2023	-	X	X	X	X
Penso 2021	+	+	+	+	+
Penso 2022	+	+	+	+	+
Popensu 2021	-	X	-	-	X
Qin 2020	+	+	+	+	+
Ribeiro 2023	X	X	X	X	X
Song 2021	+	+	+	+	+
Sulaiman Vesal 2020	+	-	-	-	-
Tran 2020	+	-	-	-	-
Vesal 2021	+	+	+	-	-
Wang 2023	-	+	+	-	-
Wang and Zhang 2021	-	+	+	+	-
Xue 2021	+	+	+	+	+
Yalcinkaya 2021	-	+	+	+	-
Yan 2022	+	+	+	+	+
Yan Wang 2021	-	+	+	+	-
Yi Wang 2022	+	+	+	+	+
You 2021	+	X	X	X	X
Zarvani 2021	+	+	+	+	+
Zhao 2020	+	+	+	+	+
Zhensen Chen 2023	+	+	+	+	+
Alskaf 2024	+	+	+	+	+
Alskaf 2025	+	+	+	+	+
Amyar 2023	-	X	X	-	X
Baraboo 2025	+	+	+	+	+
Barón 2023	-	+	+	-	-
Ben Khalifa 2025	+	+	+	+	+
Chen 2024	+	+	+	+	+
Cockrum 2024	+	+	+	+	+
Elizar 2024	-	+	+	X	X
Hatfaludi 2024	+	-	-	-	-
Kim 2023	+	X	X	X	X
Kolk 2024	+	+	+	+	+
Righetti 2024	+	X	-	+	X
Shaaf 2023	X	-	-	-	X
Xu and Shi 2025	+	+	+	+	+
Leite 2025	+	+	+	+	+
Pham 2025	X	-	-	-	X

Risk of bias domains

Domains:
D1: Patient selection.
D2: Index test.
D3: Reference standard.
D4: Flow & timing.

Judgement
X High
- Some concerns
+ Low
