# Supplementary material for: Deep learning for cardiac MRI: performance evidence and barriers to clinical integration. A Systematic Review and Meta-Analysis
Source: Eur Heart J Imaging Methods Pract. 2026 Mar 16;4(1):qyag045. doi: 10.1093/ehjimp/qyag045 (PMC13007597; doi:10.1093/ehjimp/qyag045)
Supplement: qyag045_Supplementary_Data [file qyag045_supplementary_data.zip › Supplementray Tables.docx]

**Supplementary table 1. Summary of studies related to image segmentation**

| Author | Year | Participants | Datasets | Training set | Validation | Testing data | Network architecture | Type of training | Conclusion |
| --- | --- | --- | --- | --- | --- | --- | --- | --- | --- |
| Parikh et al. | 2023 | 150 subjects with myocardial infarction, abnormal right ventricle, dilated cardiomyopathy, and hypertrophic cardiomyopathy. | ACDC and M & M | ACDC: 100 images, M & M: 150 annotated images | Not specified | ACDC: 50 images, M & M: 200 images, | Dense Unet with bidirectional convolution LSTM | Supervised | The proposed network is effective for the segmentation of LV. |
| Khalil et al. | 2023 | 360 patients with a variety of RV and LV pathologies as well as a control group | M&Ms-2 challenge data | 160 cases | 40 cases | 160 cases | nnUnet. A late fusion multi-encoder U-Net | Supervised | The proposed network can effectively reduce segmentation failures on outlier cases, which improves overall segmentation performance. |
| Hu et al. | 2023 | 4638 cardiac cases (acute myocardial infarction, subsequent myocardial infarction, cardiomyopathy, chronic ischaemic heart disease, heart failure) from the UK Biobank | Cardiac MRI cases from the UK Biobank with ground truth available for left and RV contours | CMR images in the SA view of 1000 subjects | Remaining cases | 4638 cases were used for the analysis, with a separate test set of 600 subjects compared against another model | U-net | Supervised | DL approach can effectively segment the LV/RV from cardiac MRI datasets. |
| Wang et al. | 2023 | 49 males, 22 females | 71 CMR short axis datasets, images from 49 males and 22 females, aged 23 to 93 years, with CVDs | 42 cases | 14 cases | 15 cases | Deep Atlas Network, incorporating a Dense Multi-Scale U-net (DMU-net) | supervised | The proposed deep atlas network method outperforms other methods in segmentation accuracy, particularly in the top areas. |
| Ribeiro et al. | 2023 | Not Specified | Sunnybrook and ACDC, in addition, samples of a private dataset (InCor) | ACDC, Sunnybrook, InCor | - | ACDC, Sunnybrook, InCor | U-net | Supervised | DL produces anatomically consistent segmentations and is comparable with experts. |
| Gavirni et al. | 2023 | Not Specified | SSSIHMS dataset | 70 | 15 | 15 | U-Net-based | Supervised | The proposed DL approach enhances the performance of segmentation networks. |
| Das et al. | 2023 | Not Specified | ACDC | 1328 image/label pairs. | 172 image/label pairs | 366 image/label pairs | UNET | Supervised | The proposed model can be effective for the segmentation of CMR images. |
| Chen et al. | 2023 | 50 hypertrophic cardiomyopathy (HCM) patients | Cardiac SA MR images from the MRPEAT dataset | 2121 image slices | 20% of training data set | Patient data | Triple-stage Unet model | Supervised | The proposed model segmentation results suggest that PEAT volume is a promising biomarker for CVD diagnosis and is effective compared to other approaches. |
| Wang et al. | 2023 | 34 subjects | 4000 cardiac MR images for the dataset | 7:1:2 for training, validation, and testing. | 7:1:2 for training, validation, and testing. | 7:1:2 for training, validation, and testing. | U-Net++ network | Supervised | The proposed method demonstrated competitive performance in segmenting seven cardiac tissues from MRI data. |
| Chen et al. | 2023 | - | ACDC, MSCMRSeg, and MyoPS | ACDC | - | MyoPS | U-Net | Supervised | The proposed cardiac MRI segmentation method outperforms existing methods. |
| Akesson et al. | 2023 | 1114 subjects | Clinical scans collected between 2019 and 2020 (81%) and short-axis CMR data from previous research projects collected between 2004 and 2020 (19%). | 1114 | - | 320 | CNN | Semi-supervised | The deep learning pipeline can significantly speed up right ventricular assessments compared to manual methods, though results may vary with different RV delineation guidelines. |
| Ammann et al. | 2023 | 148 patients | - | 119 training cases (1,955 images) | - | 29 test cases (479 images | Compared three CNN architectures - U-Net, FCN, and MultiResUNet | Supervised | Although results for segmentation were comparable to experts, it is important to ensure quality assurance in medical AI for reliable cardiac MRI efficiency. |
| Yan et al. | 2022 | - | 1354 cardiac MRI | The dataset was divided into training set, validation set, and test set, but specific division ratios or sizes for each set are not provided in the excerpts. | - | - | SegNet | Supervised | The deep learning model's segmentation accuracy is sufficient for most clinical applications and supports left ventricular identification in cardiac MRI. |
| Chang et al. | 2022 | 95 participants | - | - | - | - | 2D U-net | Supervised | The DL algorithm provided automated T1 and ECV measurements that were comparable to those obtained by radiologists. |
| Penso et al. | 2022 | The study included 230 patients, with 100 having Cardiac Implanted Electronic Devices (CIED). | CMR images from a multicenter patient | 70% | 15% | 15% | CNN>> U-net | Supervised | The proposed method demonstrated promising performance in cardiac segmentation from CMR images with susceptibility artifacts. |
| Wang et al. | 2022 | 150 patients | 15000 artificially segmented images were used | 12000 images | - | 3000 images for model evaluation | UU-NET | Supervised | The UU-NET-based segmentation algorithm significantly enhances accuracy. |
| Lin et al. | 2022 | 34 subjects with chronic ischaemic cardiomyopathy​​. | - | 8430 2D image slices | - | 7250 2D image slices | CTAEM-Net >> Auto-Encoder M. (AE) net | Supervised | CTAEM-Net DL model is effective for segmentation and has demonstrated generalizability across diverse imaging datasets. |
| Arai et al. | 2022 | 150 consecutive patients | - | 1400 images from 70 patients | 600 images from 30 patients | 1000 images from 50 patients | U-Net convolutional neural network (CNN) | Supervised | Fully automated segmentation enables quick and efficient simultaneous evaluation and detection of enlargement in all four cardiac chambers. |
| Popescu et al. | 2021 | - | 155 2-dimensional LGE-CMR patient scans (1124 slices) and 246 synthetic “LGE-like” scans (1360 slices) obtained from cine CMR | 2484 images from 2 sources:  1124 2-D LGE-CMR slices from 75% of available patients  and all 1360 “LGE-like” | - | LGE-CMR images from the remaining 25% of patients  (269 2-D images) | ACSNet, consists of 3 sub-networks: 1&2: ResU-Net. 3- AE network (Auto-Encoder) | Supervised | The DL network provides expert-level LGE-CMR myocardium and scar segmentation. |
| Yalcinkaya et al. | 2021 | - | - | 96 stress/rest perfusion studies with suspected ischemia | 96 stress/rest perfusion studies with suspected ischemia | 40 independent adenosine-stress CMR perfusion studies were obtained at an external site​​. | Vanilla U-Net | Supervised | The proposed DL method enhances spatiotemporal data augmentation and outperforms the current 2D approach in generalization. |
| Wang and Zhang | 2021 | 137 patients | 137 groups of four-chamber view MRI cardiac images from 137 patients. Each group contains 18 continuous frames, encompassing contraction and diastole cardiac states​​. | 130 groups of images for training | - | 7 groups for testing | RNN | Supervised | The proposed algorithm improves cardiac state estimation by leveraging time-sequential LV segmentation. |
| Fahmy et al. | 2021 | 191 hypertrophic cardiomyopathy patients | MRI datasets were obtained from patients with hypertrophic cardiomyopathy, including both short-axis LGE and cine sequences planned using the same reference scan​​ | 50%, 81 patients | 25%, 40 patients | 25%, 41 patients | CNN | Supervised | CNN-based LGE-Cine fusion can enhance the accuracy of automated scar quantification. |
| Vesal et al. | 2021 | 145 patients with various pathologies, including myocardial hypertrophy, regional-wall motion abnormalities, atrial septal defect, mildly enlarged LV, LV dysfunction, etc. | STACOM LVQuan 2018 challenge training dataset | 81 patients | 40 patients | 41 patients subsets, with an external testing dataset including 29 patients | UNet | Supervised | The proposed segmentation method is effective across different cardiac morphologies, and low-contrast MR sequences. |
| Wang et al. | 2021 | 95 volumes from cardiac MRI studies, detailed patient demographics or the exact number of participants are not directly mentioned​​. | MICCAI 2009 LV and 2012 RV segmentation challenges, | Utilized 95 volumes (1076 slices) with approximately 80% as for training datasets | - | ~20% as for testing datasets​​ | U-Net | - | The proposed fully automatic segmentation method for the LV endocardium and epicardium from MRI is accurate. |
| Song et al. | 2021 | - | ACDC | 100 labeled subjects (including 1902 image  slices) | - | 20 subjects (containing 380 slices) | LCC-Net’s | Semi supervised | The LCC-Net is effective for segmentation. |
| Ammar et al. | 2021 | 150 patients | ACDC | 100 cases | - | 50 cases | U-net | Supervised | DL is effective for cardiac structure segmentation. |
| Zarvani et al. | 2021 | - | Sunnybrook Cardiac Data | 568 | - | 142 | Residual Network of Residual Network, ROR‑Unet | Supervised | Effective for LV segmentation. |
| Galea et al. | 2021 | - | ACDC, imATFIB | 100 volumes | 100 volumes | 50 volumes | U-Net, DeepLabV3+ | Semi supervised | DL algorithm is effective for cardiac structure segmentation. |
| Penso et al. | 2021 | 210 patients | Two datasets were used: DB1, consisting of 210 patients with hypertrophic cardiomyopathy (HCM), dilated cardiomyopathy (DCM), and ventricular arrhythmias (VA), and DB2, consisting of 12 healthy volunteers. | 70% | 15% | 15% | U-net | Supervised | DL methods, especially with dense skip connections, provide accurate and efficient LV and RV contour segmentation. |
| Bartoli et al. | 2020 | - | - | 272 | 27 | 150 | DenseNet architecture | Supervised | DL framework provides more consistent and accurate segmentation of cardiac trabeculations compared to inter- and intraobserver analyses. |
| Vesal et al. | 2020 | - | ACDC STACOM, LASC STACOM | 80% | - | 20% | A 3D dilated residual U-Net (3D DR-UNet) | Supervised | The proposed DL approach is robust and capable of producing accurate high-resolution segmentations even amidst varying pathologies, low contrast, and noise in CMR volumes. |
| Graves et al. | 2020 | - | InCor (INC), Sunnybrook (SUN), ACDC (ACD), LVQuan19 (LVQ), LVSC-2011 (LVS) | 70% | 15% | 15% | U-net | Supervised | The proposed DL network is effective for LV segmentation. |
| Abdeltawab et al. | 2020 | - | ACDC MICCAI challenge 2017 | 100 subjects | - | 50 subjects | FCN, fully convolutional neural network | Supervised | The proposed DL segmentation approach effectively generalizes across various datasets. |
| Tran et al. | 2020 | 100 individuals | 5,729 short-axis cine CMR slices | 50 individuals | 10 individuals | 40 individuals | U-Net |  | DL-based method effectively automates RV segmentation. |
| Qin et al. | 2020 | 30 patients | 86 MR slices, with each slice containing 73 images​​. | 46 slices | 10 slices | 30 slices | Dense V-Net | Supervised | The DenseV-Net method surpasses traditional convolutional networks like V-Net, UNet, and FCN in automating LV segmentation. |
| Zhao et al. | 2020 | ASD patients | 200 MRI slices | Not provided | - | 200 MRI slices | U-Net | Supervised | The proposed segmentation technique is effective for accurate evaluation of atrial septal defects. |
| Luo et al. | 2020 | - | ACDC | 1020 MRIs | 340 MRIs | 340 MRIs | U-Net | Supervised | The proposed method is effective for segmentation. |
| Du et al. | 2020 | - | STACOM 2018 challenge dataset | 80 subjects | - | 20 subjects | - | Supervised | The proposed DL method’s performance in left atrium segmentation and visualization suggests it could enhance clinical diagnosis and treatment of atrial fibrillation. |
| Liu et al. | 2020 | - | ACDC | 100 patients | - | 50 patients | Residual CNN, also integrates U-Net as a submodule within the framework​​. | Supervised | The proposed DL methods is effective for cardiac segmentation and disease diagnosis. |
| Baraboo et al. | 2025 | 44 patients | Internal dataset with RTPC MRI; 15,307 semi-manual contours used | 34 patients | - | 10 patients | U-Net | Supervised | CNN achieved human-comparable LA segmentation performance on RTPC MRI; enabled fully automated LA flow quantification with high agreement in derived parameters; robust to heart rate variability. |
| Ben Khalifa et al. | 2025 | 163 patients | Custom dataset of LGE CMR images | 1389 images | - | 168 images | U-Net | Supervised | The VGG16-MLP model achieved 96% accuracy in classifying MI, myocarditis, and healthy tissue from LGE CMR, outperforming other models and human readers. |
| Elizar et al. | 2024 | 150 patients | ACDC | 100 training cases | - | 50 testing cases | DeSPPNet | Supervised | DeSPPNet achieved high segmentation accuracy for cardiac organs (LV, RV, myocardium) with best Dice = 0.859, IoU = 0.800, and overall pixel accuracy = 0.993. |
| Kim et al. | 2023 | 83 patients | Multi-institutional CMR datasets from two centers | - | Entire cohort of 83 subjects used | - | U-Net | Semi supervised | DL-based software achieved strong segmentation performance and non-inferior T2 value measurement compared to manual references. |
| Leite et al. | 2025 | 648 patients | - | 482 patients | 142 patients | 60 patients | ResUnet | Supervised | The proposed model resulted in fully automated and reliable LV and LA strain measures, reaching human reproducibility. |
| Pham et al. | 2025 | 45 patients | ACDC | 70 cases | 10 cases | 20 cases | CapNet | Supervised | The promising evaluation metrics show comparative results in both Dice and IoU indices compared to SOTA CNN-based and Transformer-based architectures. |

**Supplementary table 2. Summary of studies related to image diagnosis and prediction**

| Author | Year | Participants | Datasets | Training set | Validation | Testing data | Network architecture | Type of training | Conclusions |
| --- | --- | --- | --- | --- | --- | --- | --- | --- | --- |
| Gao et al. | 2023 | 329 patients diagnosed with heart failure with reduced ejection fraction | Patients underwent routine CMR examinations between January 2015 and April 2020, and electronic health record data were collected alongside clinical demographic information, laboratory data, and electrocardiographic information. | Not explicitly mentioned; the study design is retrospective, indicating the use of existing patient data. | Internal validation was conducted using a bootstrap technique | Patient data | R Fast CNN | supervised | DL model effectively predicts outcomes in HFrEF patients, outperforming conventional methods in prediction efficiency. |
| Diao et al. | 2023 | 302 LVH patients | 302 LVH patients | 191 patients | 48 patients | 63 patients | based on Res-Unet model | supervised | The DL model can help diagnose the cause of left ventricular hypertrophy and aiding clinical decisions. |
| Agibetov er al. | 2021 | 502 patients | CMR data from 502 patients | 1000 epochs | - | - | CNN | Supervised | DL is effective for diagnosis of cardiac amyloidosis. |
| Xue et al. | 2021 | - | Cine late gadolinium enhancement (LGE) and T1 mapping examinations from two hospitals | 2329 patients (34 089 images | - | 7723 images from 531 consecutive patients ( | CNN | Supervised | The CNN model was effective for detection on various CMR image sequences. |
| You et al. | 2021 | 37 patients were selected for the study, and each sequence contains 25 frames​​. | The CMR datasets of the normal heart and patients with hypertrophic obstructive cardiomyopathy (HOCM) were used for the study​​. | The ratio of training set to test set is 3:1​​ | - |  | A double-branch neural network CMR-based HOCM recognition algorithm was implemented, compared with traditional classification algorithms such as ResNet and DenseNet​​. | Supervised | The DL method effectively captures heart morphology and motion, enhancing recognition accuracy. |
| Diller et al. | 2020 | 372 patients | - | 42 patients |  | - | U-Net | Supervised | The DL can efficiently estimate prognosis in patients with Tetralogy of Fallot. |
| Alskaf et al. | 2024 | 1286 patients | Retrospective dataset from SP-CMR scans and Late gadolinium enhancement (LGE) | 772 patients | 193 patients | 322 patients | CNN | supervised | SP-CMR images can predict mortality in CAD patients without clinical data; prediction improves with HNN combining image + clinical data (AUC: CNN = 72%, HNN = 82%) |
| Alskaf et al. | 2025 | 2740 patients | Dataset from stress perfusion CMR studies | 1918 patients | 411 patients | 411 patients | CNN | supervised | This hybrid DL approach enhances risk stratification in CAD. |
| Amyar et al. | 2023 | 3000 patients | Cine CMR images from Weill Cornell Medicine | 2000 patients | 500 patients | 500 patients | Spatiotemporal Residual Attention Network (ST-RAN) with factorized 4D convolution (Conv3Plus1D) | supervised | The ST-RAN model detects myocardial scar using contrast-free cine MRI in both ischemic and non-ischemic diseases, outperforming other DL methods. |
| Barón et al. | 2023 | - | Multi-institutional cardiac MRI datasets | 80/20 train/test split and perform five-fold cross-validation on the training dataset. | - | - | U-Net | supervised | U-Net++ techniques enhance LVNC detection. |
| Chen et al. | 2024 | 215 patients | Cine cardiac MRI (short-axis view) datasets from two hospitals | 172 images | - | 43 images | CNN | Semi-supervised | The model showed strong generalization across two centers and supports AI-assisted diagnosis in LVH conditions. |
| Cockrum et al. | 2024 | Development cohort: 807 patients  External validation cohort: 157 patients | Internal dataset from a large single-state health system | 565 patients | 157 patients | 242 patients | Vision transformer (ViT) | supervised | The ViT model achieved high diagnostic performance in distinguishing cardiac amyloidosis from HCM and other conditions using cine and LGE CMR. |
| Hatfaludi et al. | 2024 | 269 patients | Single center dataset | - | - | - | CNN | supervised | The DL model accurately distinguished myocarditis from healthy cases. |
| Kolk et al. | 2024 | 289 patients | Short-axis LGE CMR scans 12-lead ECGs Clinical data | - | - | 103 external testing cohort | Residual Variational Autoencoder (rVAE) for LGE-MRI & ECG | supervised | The DEEP RISK model combining CMR, ECG, and clinical data outperformed individual modalities with an AUC of 0.84, sensitivity 98%, and specificity 73%, showing high predictive accuracy for ventricular arrhythmia risk in non-ischemic cardiomyopathy patients pre-ICD. |
| Righetti et al. | 2024 | 206 patients | Cine CMR data | 164 patients | 25 patients | 42 patients | CNN | supervised | CNN achieved strong scar detection results without contrast. |
| Shaaf et al. | 2023 | 45 patients | Sunnybrook cardiac dataset | 80% for training | - | 20% for testing | R Fast CNN | supervised | The proposed model was adequate, with accuracy, precision, recall, and F1 score values of 0.91, 0.94, 0.95, and 0.95, respectively. |
| Xu and Shi | 2025 | 132 patients | Sunnybrook cardiac dataset | 80% for training | - | 20% for testing | U-Net | supervised | Integrating radiomic features into a deep learning segmentation pipeline improves MI detection and interpretability in cine-CMR. |
