# Supplementary figures and images for: Deep learning for cardiac MRI: performance evidence and barriers to clinical integration. A Systematic Review and Meta-Analysis
Source: Eur Heart J Imaging Methods Pract. 2026 Mar 16;4(1):qyag045. doi: 10.1093/ehjimp/qyag045 (PMC13007597; doi:10.1093/ehjimp/qyag045)

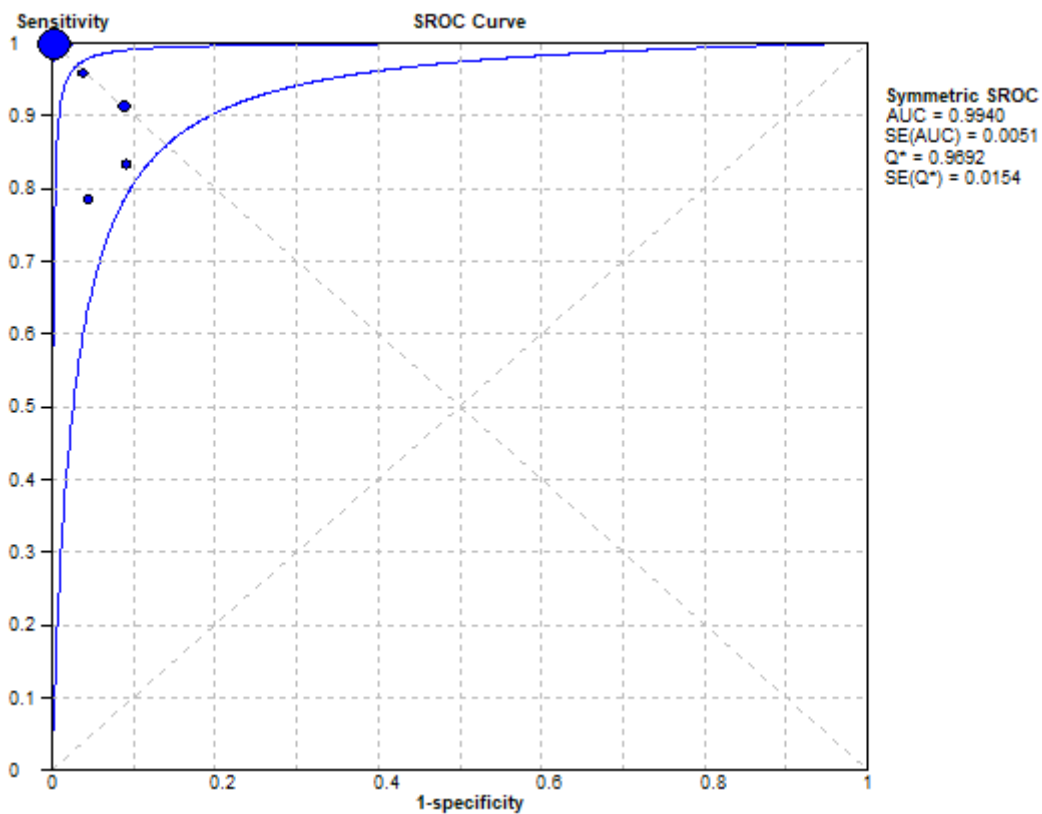

Supplement: qyag045_Supplementary_Data [file qyag045_supplementary_data.zip › Supplemantray figure 2.pdf]

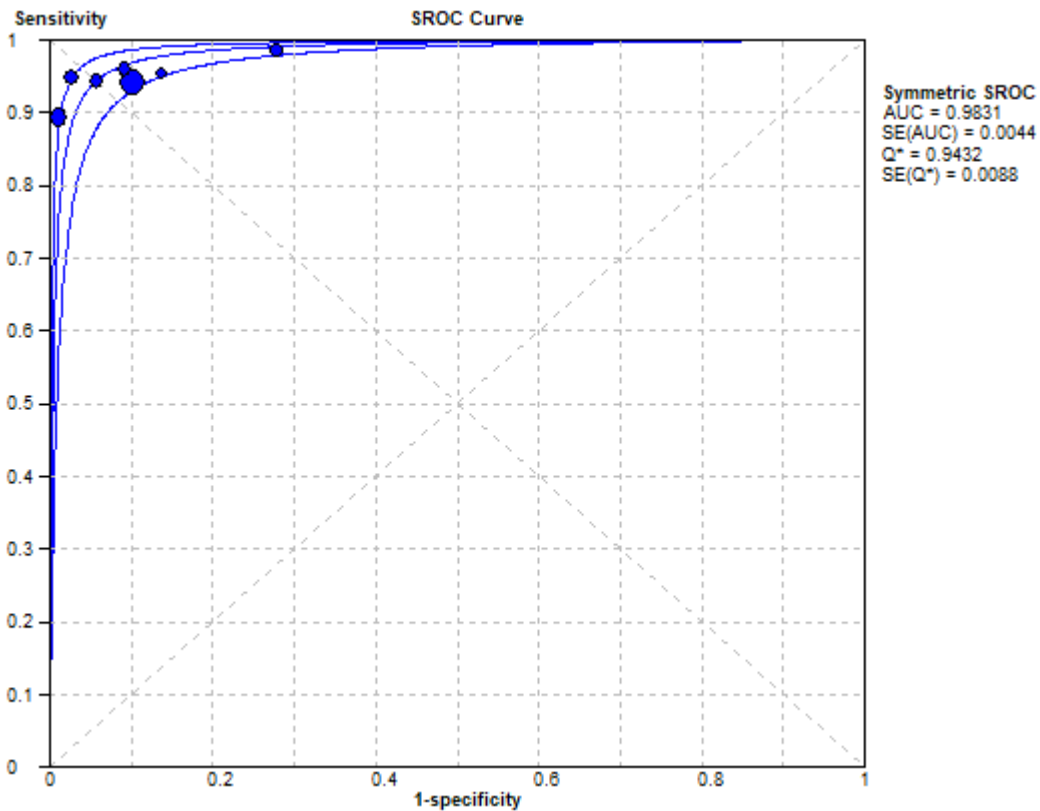

Supplement: qyag045_Supplementary_Data [file qyag045_supplementary_data.zip › Supplementray figure 1.pdf]
